# Effects of FGFR Tyrosine Kinase Inhibition in OLN-93 Oligodendrocytes

**DOI:** 10.3390/cells10061318

**Published:** 2021-05-25

**Authors:** Ranjithkumar Rajendran, Gregor Böttiger, Niklas Dentzien, Vinothkumar Rajendran, Bischand Sharifi, Süleyman Ergün, Christine Stadelmann, Srikanth Karnati, Martin Berghoff

**Affiliations:** 1 Experimental Neurology, Department of Neurology, Justus Liebig University of Giessen, 35385 Giessen, Germany; ranjithkumar.rajendran@neuro.med.uni-giessen.de (R.R.); gregor.w.boettiger@med.uni-giessen.de (G.B.); niklas.dentzien@med.uni-giessen.de (N.D.); vinothkumar.rajendran@neuro.med.uni-giessen.de (V.R.); bischand.sharifi@med.uni-giessen.de (B.S.); 2 Institute of Anatomy and Cell Biology, University of Würzburg, Koellikerstrasse 6, 97080 Würzburg, Germany; sueleyman.erguen@uni-wuerzburg.de (S.E.); srikanth.karnati@uni-wuerzburg.de (S.K.); 3 Institute for Neuropathology, Clinic for Neurology, University Medical Center, 37075 Göttingen, Germany; cstadelmann@med.uni-goettingen.de

**Keywords:** multiple sclerosis, oligodendrocytes, dovitinib, AZD4547, FGFR signaling, myelin

## Abstract

Fibroblast growth factor (FGF) signaling is involved in the pathogenesis of multiple sclerosis (MS). Data from neuropathology studies suggest that FGF signaling contributes to the failure of remyelination in MS. In MOG_35–55_-induced EAE, oligodendrocyte-specific deletion of *FGFR1* and *FGFR2* resulted in a less severe disease course, reduced inflammation, myelin and axon degeneration and changed FGF/FGFR and BDNF/TrkB signaling. Since signaling cascades in oligodendrocytes could not be investigated in the EAE studies, we here aimed to characterize FGFR-dependent oligodendrocyte-specific signaling in vitro. FGFR inhibition was achieved by application of the multi-kinase-inhibitor dovitinib and the FGFR1/2/3-inhibitor AZD4547. Both substances are potent inhibitors of FGF signaling; they are effective in experimental tumor models and patients with malignancies. Effects of FGFR inhibition in oligodendrocytes were studied by immunofluorescence microscopy, protein and gene analyses. Application of the tyrosine kinase inhibitors reduced FGFR1, phosphorylated ERK and Akt expression, and it enhanced BDNF and TrkB expression. Furthermore, the myelin proteins CNPase and PLP were upregulated by FGFR inhibition. In summary, inhibition of FGFR signaling in oligodendrocytes can be achieved by application of tyrosine kinase inhibitors. Decreased phosphorylation of ERK and Akt is associated with an upregulation of BDNF/TrkB signaling, which may be responsible for the increased production of myelin proteins. Furthermore, these data suggest that application of FGFR inhibitors may have the potential to promote remyelination in the CNS.

## 1. Introduction

Demyelination of the central nervous system (CNS) causes degeneration of axons [1] associated with permanent disability [2,3,4]. The physiological response to demyelination is robust and leads to effective remyelination [5]. However, remyelination often fails in inflammatory demyelinating diseases such as MS [2,6,7]. The pivotal impediment for remyelination in MS is most likely not the recruitment of oligodendrocyte progenitor cells (OPCs) to lesions, but OPCs` inability to differentiate into mature myelin-producing oligodendrocytes (OLs) [8,9]. Growth factors have been associated with the differentiation of oligodendrocytes in demyelinating pathologies. These include brain derived neurotrophic factor (BDNF), nerve growth factor (NGF), insulin-like growth factor (IGF), platelet derived growth factor (PDGF), ciliary neurotrophic factor (CNTF) and FGF [10,11]. Molecular analyses of brain tissue suggest that FGF/FGF receptor (FGFR) signaling pathways are relevant to the pathogenesis of MS. In lesion areas, FGF1 is expressed in oligodendrocytes, astrocytes, microglia/macrophages and infiltrating lymphocytes [12]. FGF2 is mainly found in microglia/macrophages [13], whereas FGF9 is expressed in oligodendrocytes and astrocytes [14]. Further, the corresponding FGFR1 is upregulated in an OPC subpopulation in lesions [13]. Moreover, in the cerebrospinal fluid (CSF), FGF2 levels were increased in patients with MS, and their highest expression was found in relapse [15].

MOG_35–55_-induced experimental autoimmune encephalomyelitis (EAE) is the most widely used animal model of MS. We recently investigated the role of oligodendrocyte-specific deletion of *FGFR1* and *FGFR2* in EAE [16,17]. Cell-specific deletion of these genes decreased disease severity, reduced lymphocyte and macrophage/microglia infiltration, and resulted in less myelin and axon degeneration. Furthermore, changes in FGF/FGFR downstream signaling and the BDNF/TrkB pathway were induced by oligodendrocyte-specific FGFR deletions. Based on these findings of oligodendrocyte FGFR function on inflammation and neurodegeneration, we sought to characterize the mechanisms responsible for demyelination in particular with respect to myelin expression in oligodendroglia.

We hypothesized that in vitro application of FGFR inhibitors results in reduced proliferation, enhanced differentiation and upregulation of myelin associated proteins in OLs. To test our hypothesis, we used OLN-93 cells and treated them with AZD4547 and dovitinib. AZD4547 is a novel FGFR1-3 inhibitor with high oral bioavailability, selectivity and potency [18] down-regulating RAS-MAPK-ERK and phosphoinositide 3-kinase (PI3K)-Akt pathways [19], which was employed in Phase II studies for treatment of different cancers. The favorable side effect profile of AZD4547 makes it an auspicious candidate for inhibition of FGFR1-3 in humans [20,21]. Dovitinib is a multi-kinase inhibitor targeting FGFR1-3 along with vascular endothelial growth factor (VEGF), platelet-derived growth factor receptor (PDGFR) and tyrosine kinases of Class III (KIT, RET) [22]. It is a well-known tyrosine kinase inhibitor that has been trialed in clinical studies for its effect on various cancers. Other than AZD4547, dovitinib targets a broader spectrum of receptors, and may therefore, provide insights on the role of complementary growth factor receptors. Overall, receptor-targeted modulation of FGFR signaling has yielded promising results in cancer disease models [23,24] and potent inhibitors of FGFRs are currently tested in cancer treatment trials [25].

Administration of these FGFR tyrosine kinase inhibitors resulted in less FGFR1 expression and less phosphorylation of Akt and ERK. Interestingly, AZD4547 and dovitinib both promoted the upregulation of TrkB/BDNF and enhanced expression of myelin proteins. Thus, this study revealed that AZD4547 and dovitinib are efficacious in inducing differentiation of OLN-93 oligodendrocytes and the promotion of myelination in vitro. The findings presented here support the view of FGFR inhibition as a therapeutic tool to enable remyelination in MS.

## 2. Materials and Methods

### 2.1. Cell Culture

The permanent oligodendroglia cell line OLN-93 was kindly provided by Prof. Markus Kipp, University of Rostock, Germany. Cells were cultured in growth medium consisting of Dulbecco’s Modified Eagle Medium (DMEM) (Gibco, Invitrogen, Carlsbad, CA, USA) supplemented with 10% heat inactivated FBS (Gibco, Invitrogen, Carlsbad, CA, USA) and 1% penicillin/streptomycin (Life Technologies Limited, Renfrew, UK). Cells were maintained at 37 °C in an incubator (Sanyo, Osaka, Japan) at a controlled atmosphere (95% humidity, 5% CO_2_). At a confluence of at least 80%, cells were sub-cultured. Washing with PBS (PAN Biotech, Aidenbach, Germany) was followed by cell detachment using 0.5% Trypsin-EDTA (Gibco, Invitrogen, Carlsbad, CA, USA), centrifugation of detached cells and resuspension in fresh medium. Experiments were performed at passage 8–20.

### 2.2. Experimental Treatment of Cells

Four groups were deployed and treated with either dovitinib (Selleck Chemicals, Houston, TX, USA), AZD4547 (Selleck Chemicals, Houston, TX, USA), FGF2 (R&D Systems, Minneapolis, MN, USA) or utilized without treatment as a control. AZD4547 and dovitinib were dissolved in dimethyl sulfoxide (DMSO) (Carl Roth GmbH, Karlsruhe, Germany) and stored at −20 °C until use. Compounds were applied at a concentration of 1 µM (AZD4547, dovitinib) or 25 ng/mL (FGF2). DMSO controls have been included in all experiments. The inhibitors were applied at an effective concentration as previously established [18,26]. All treatments were added to DMEM growth medium and setups were incubated for 24 h, after which appropriate amounts of cells were taken for further analysis.

### 2.3. Proliferation Measurement

To assess cell proliferation under different treatment conditions several assays were utilized. Cells were grown in DMEM culture medium supplemented with 10% FBS and the respective compounds at humidified conditions. After 24 h of incubation manual counting was performed using a Neubauer improved chamber (Karl Hecht “Assistant”, Altnau, TG, Switzerland) and Trypan blue dye (Carl Roth, Karlsruhe, Germany) as described by others [27]. For photometric evaluation of proliferation, cells were seeded at 2 × 10^4^ cells/well in a 96 well plate (Greiner Bio-One, Frickenhausen, Germany) before treatment. After 24 h of treatment, a WST-1 assay (Roche Applied Science, Mannheim, Germany) was carried out as described by manufacturer. Absorption was measured by ELISA-Reader (Multiscan EX, Thermo Fisher Scientific, Langenselbold, Germany) at a wavelength of 405 nm using a reference wavelength of 492 nm.

### 2.4. Cytotoxicity Assessment

Possible cytotoxic effects of applied compounds were studied by measuring lactate dehydrogenase (LDH) in the supernatant of incubated cells (96 well plate, 0.5 × 10^4^ cells/well) using a prefabricated kit (Cytotoxicity Detection Kit (LDH), Roche Diagnostics, Mannheim, Germany). Cell plates were centrifuged for 10 min at 250× *g* and supernatant was used to measure absorbance at 492 nm (reference wavelength: 620 nm) to calculate cytotoxicity as described by manufacturer.

### 2.5. Western Blotting and Antibodies

For protein extraction, cells were incubated in T-75 flasks for 24 h as described above. Media was then removed, and cells were washed with PBS. After centrifugation, buffer was removed, cells were lysed and protein concentration was quantified in a nanophotometer (Implen GmbH, Munich, Germany) according to the manufacturer’s instructions. After normalization, proteins were separated by 10% SDS-PAGE gel electrophoresis. Separated protein was transblotted to a nitrocellulose membrane (GE Healthcare, Amersham™ Protran™, Buckinghamshire, UK) and after blocking with 5% BSA (Merck, Darmstadt, Germany) primary antibodies were applied in an appropriate dilution in tris buffered saline with tween 20 (TBST) with 5% BSA and incubated overnight. The antibodies used in this study can be found in Supplementary (Supplementary Table S1). SuperSignal™ West Pico chemiluminescent substrate (Thermo Fisher Scientific, Waltham, MA, USA) was used to visualize the protein bands imaged in an ECL Chemocam Imager (Intas-Science, Göttingen, Germany) and quantified using ImageJ software (NIH, Stapleton, NY, USA).

### 2.6. RNA Analysis

For RNA extraction, cells were incubated in T-75 flasks for 24 h as described above. Media was then removed, and cells were washed with PBS. After centrifugation, buffer was removed and the cellular RNA extracted with peqGOLD Total RNA Kit as described by the manufacturer (VWR, PEQLAB, Darmstadt, Germany). Total amount of RNA was quantified in a nanophotometer and 1 µg of RNA was used for subsequent cDNA synthesis with QuantiTect Reverse Transcription Kit (Thermo Fisher Scientific, QIAGEN, Hilden, Germany). All samples were diluted in 200 µL RNAse free H_2_O from which 1 µL was used for comparative quantitative real time PCR (qRT-PCR) MicroAmp^®®^ Fast Reaction Tubes (Applied Biosystems, Darmstadt, Germany). A total 7 µL of H_2_O, 1 µL of forward and reverse primer (FP and RP, respectively) and 10 µL of SYBR^®®^ Green qPCR Supermix (Bio-Rad, Hercules, CA, USA) was added. Primers for *FGFR1*, *BDNF*, *TrkB*, PLP, *CNPase*, *SEMA3A*, *TGFβ* and *GAPDH* as internal control were purchased from Eurofins Genomics (Ebersberg, Germany). The primers used in this study can be found in Supplementary Table S2. PCR was performed in StepOne^®®^ Real-Time PCR system (Applied Biosystems, Darmstadt, Germany) using StepOne™ Software v2.3 (Applied Biosystems, Darmstadt, Germany) in 40 repetitive cycles: Denaturation at 95 °C for 15 s followed by annealing at 60 °C for 1 min. The three biological replicates performed contained negative controls without template cDNA and were executed in technical duplicates. Quantification of the results was carried out using the 2^−ΔΔCt^ method [28].

### 2.7. Immunofluorescence

To address the morphological alterations induced by FGFR inhibition, immunofluorescence was performed. OLN-93 cells were seeded on sterilized coverslips (R. Langenbrinck, Emmendingen, Germany) in 24-well-plates (GreinerBioOne, Frickenhausen, Germany) at a final density of 6 × 10^4^ cells/well and treated according to their assigned group as described in section experimental treatment of cells. Cells were washed with PBS, fixed using 4% paraformaldehyde and incubated in PBS containing 0.5% Triton X-100 (Sigma-Aldrich, Steinheim, Germany) for permeabilization. After blocking in PBS with 5% BSA, cells were incubated overnight at 4 °C with primary antibodies, washed and stained with respective fluorescent secondary antibodies (as listed in Supplementary Table S1). Negative controls omitting the secondary antibody were included for each treatment. Cells were counter-stained with DAPI (Carl Roth, Karlsruhe, Germany) and subsequently mounted onto microscope slides using Fluorescence Mounting Medium (DAKO, Agilent, CA, USA). Images were taken with Axioplan 2 Fluorescence Microscope (Carl Zeiss, Jena, Germany) at a magnification of 50× and 400× using ZEN software for microscope (Zen 2.3, ZEISS, Jena, Germany) for image acquisition and processing. For counting of positive cells, 10 areas on every slide were randomly selected and a total of 30 cells was counted in each spot at 50× magnification regarding the intensity and location of signal. The total number of cells was determined by the DAPI counterstain.

### 2.8. Statistical Analysis

All experiments were conducted at least in 3–6 replicates. Data were analyzed using Sigmaplot 14 (Systat, San Jose, CA, USA). Depending on distribution of data, differences between groups were analyzed either by using ANOVA or Kruskal–Wallis ANOVA followed by a Tukey post hoc test or by Dunn’s Method. *p* < 0.05 was considered statistically significant (*/^#^ ≙ *p* < 0.05, **/^##^ ≙ *p* < 0.005, ***/^###^ ≙ *p* < 0.001). Values are shown as mean ± standard error of the mean (SEM).

## 3. Results

### 3.1. FGFR Inhibition Reduces Proliferation of OLN-93 Cells

**Figure 1:**
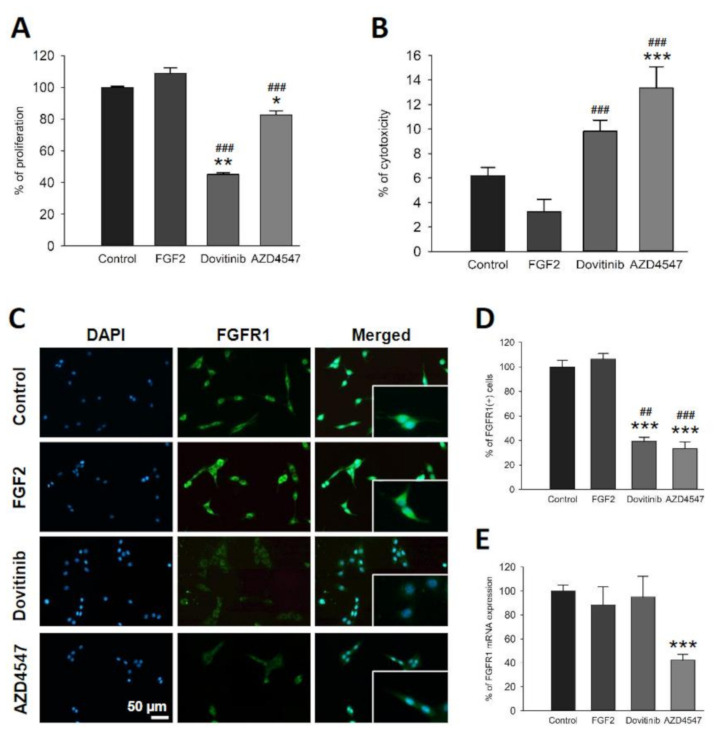
Cell viability (**A**,**B**) and FGFR expression (**C**–**E**) of OLN-93 cells incubated with dovitinib, AZD4547 or FGF2 for 24 h. Both FGFR inhibitors induced anti-proliferative responses in OLN-93 cells (**A**), while only AZD4547 was cytotoxic to these cells (**B**). Representative sections of immunofluorescence staining (**C**). Quantitative analysis of immunofluorescence staining revealed reduced numbers of FGFR1^+^ cells after dovitinib and AZD4547 treatment (**D**). FGF2 did not affect the number FGFR1^+^ cells. qRT-PCR showed downregulation of FGFR (*FGFR1*) mRNA only in cells treated with AZD4547 (**E**). Data are presented as mean ± SEM. * *p* < 0.05, ** *p* < 0.005, *** *p* < 0.001 vs. control; ^##^ *p* < 0.005, ^###^ *p* < 0.001 vs. FGF2.

To study whether AZD4547 and dovitinib alter proliferation, OLN-93 cells were treated with these inhibitors. AZD4547 and dovitinib resulted in less proliferation of OLN-93 cells compared to untreated cells (dovitinib vs. control: *p* = 0.002; AZD4547 vs. control: *p* = 0.012; dovitinib vs. FGF2: *p* < 0.001; AZD4547 vs. FGF2: *p* < 0.012) (Figure 1A). In contrast, application of FGF2 did not affect proliferation of OLN-93 cells (Figure 1A).

### 3.2. Inhibition of FGFR Is Cytotoxic to OLN-93 Cells

As these compounds inhibit growth factor receptors involved in cell survival, we investigated possible cytotoxic effects on OLN-93 cells by means of a LDH assay. Both substances were deployed at a concentration of 1 µM throughout the experiment. LDH levels in the supernatant of the treated cells revealed a significant cytotoxic effect of AZD4547 (*p* < 0.001), while dovitinib did not exert cytotoxicity in comparison to control (Figure 1B).

### 3.3. FGFR1 Expression Is Reduced by FGFR Inhibition

In the present study we investigated specific effects of pharmacological FGFR inhibition in oligodendrocytes by immunostaining and comparative qRT-PCR, after previously characterizing the effects of genetic *FGFR* silencing in OLs in the EAE model [16,17]. Application of AZD4547 and dovitinib resulted in a significant reduction of FGFR1^+^ cells compared to control (*p* < 0.001) (Figure 1C,D). FGF2 did not affect FGFR1 expression on cellular or mRNA levels (Figure 1C–E). While dovitinib treatment decreased the overall number of FGFR1^+^ cells, it did not affect *FGFR1* mRNA expression. Cells treated with AZD4547 expressed less *FGFR1* mRNA (*p* = 0.009) (Figure 1E).

### 3.4. FGFR Inhibition Decreased Phosphorylation of FGFR Downstream Signaling via Akt and ERK

**Figure 2:**
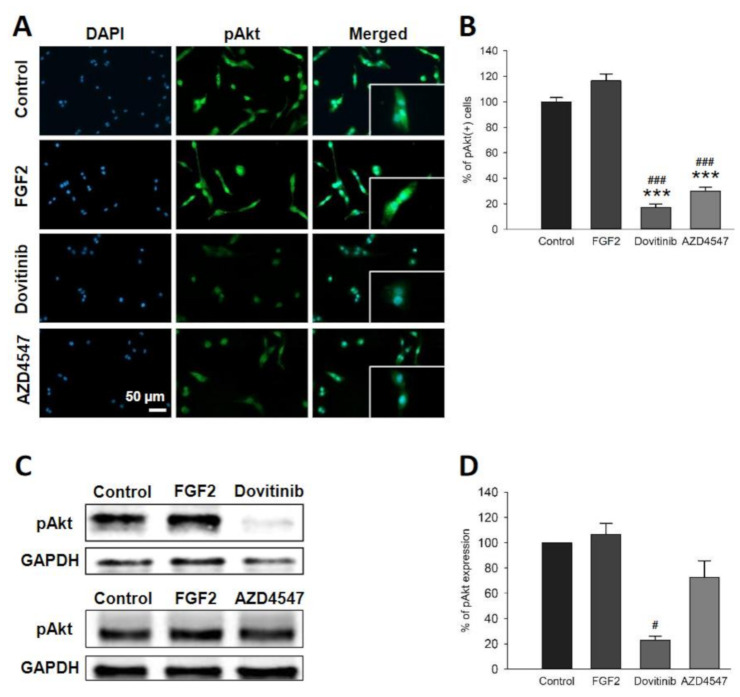
Cellular distribution (**A**,**B**) and protein expression (**C**,**D**) of pAkt. Representative sections of immunofluorescence staining (**A**). Quantification of fluorescence exposed a reduction in pAkt^+^ cell count for both inhibitors to less than 60% (**B**). Representative Western blot images of pAkt analysis (**C**). pAkt levels as quantified by Western blot (**D**). Data are presented as mean ± SEM. *** *p* < 0.001 vs. control; ^#^ *p* < 0.05, ^###^ *p* < 0.001 vs. FGF2.

**Figure 3:**
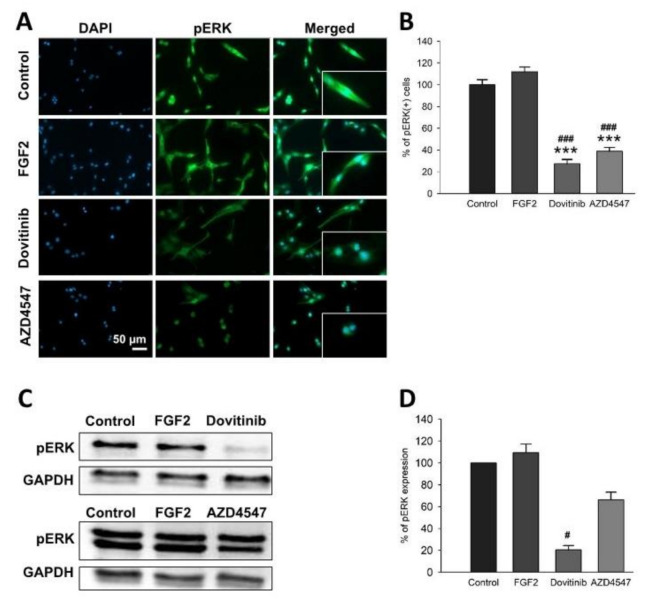
Cellular staining (**A**,**B**) for pERK and its protein expression (**C**,**D**). Representative sections for distribution and prevalence of pERK (**A**). The number of pERK^+^ cells was reduced by more than 40% in all groups treated with FGFR inhibitors (**B**). Representative images of pERK Western blot (**C**). pERK protein levels were reduced by dovitinib (**D**). Data are presented as mean ± SEM. *** *p* < 0.001 vs. control; ^#^ *p* < 0.05, ^###^ *p* < 0.001 vs. FGF2.

After studying the proliferative and cytotoxic effects of AZD4547 and dovitinib, we investigated whether these substances might be involved in FGFR signaling. For this purpose, we conducted Western blot and immunofluorescence staining for pERK and pAkt to examine the relative activation of these pathways following FGFR inhibition. The number of pAkt^+^ cells was significantly reduced by AZD4547 and dovitinib (*p* < 0.001) (Figure 2A,B). We observed a trend towards lower abundance for dovitinib compared to control (*p* = 0.131) (Figure 2C,D). Fewer pERK^+^ cells were detectable by immunofluorescence after treatment with AZD4547 or dovitinib (*p* < 0.001) (Figure 3A,B). pERK protein levels were unchanged (Figure 3C,D).

### 3.5. The Expression of Neuroprotective BDNF and Its Receptor TrkB Is Elevated Following Inhibition of FGFR Signaling

**Figure 4:**
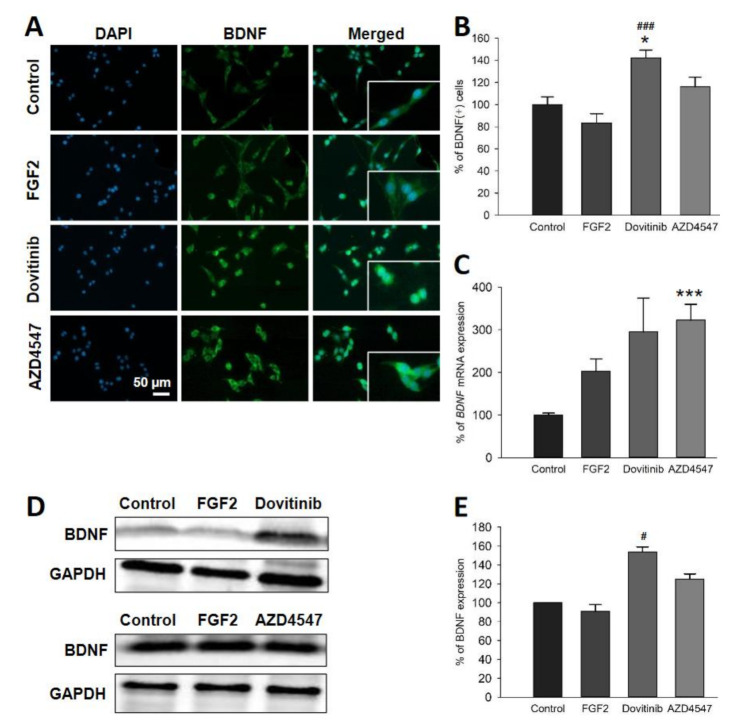
Cellular distribution (**A**,**B**), mRNA expression (**C**) and total amount of BDNF protein (**D**,**E**). Representative images are shown for immunofluorescence staining (**A**). Quantification of fluorescence showed a 40% enhancement of BDNF^+^ cell numbers by dovitinib (**B**). BDNF (*BDNF*) mRNA was abundant after inhibition with AZD4547 (**C**). Representative Western blot bands of BDNF protein expression (**D**). BDNF protein expression was increased by dovitinib (**E**). Data are presented as mean ± SEM. * *p* < 0.05, *** *p* < 0.001 vs. control; ^#^ *p* < 0.05, ^###^ *p* < 0.001 vs. FGF2.

**Figure 5:**
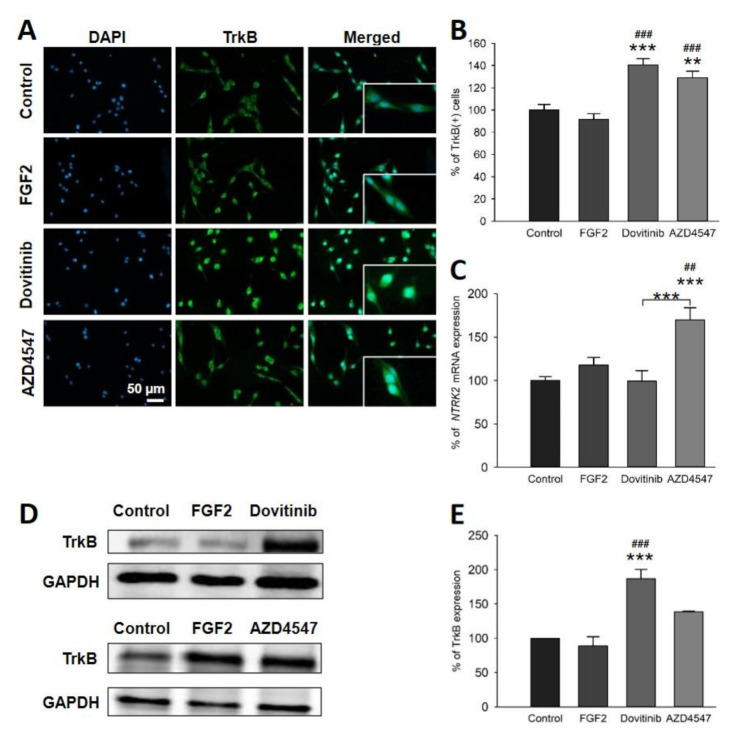
Number of TrkB^+^ cells (**A**,**B**), TrkB mRNA (**C**) and protein levels (**D**,**E**). Representative sections of immunofluorescence staining (**A**). Analysis of immunofluorescence staining revealed increased numbers of TrkB^+^ cells by dovitinib and AZD4547 of more than 20% (**B**). qRT-PCR showed an upregulation of TrkB (*NTRK2*) mRNA only in cells treated with AZD4547 (**C**). Representative Western blot images for TrkB (**D**). TrkB protein levels were increased by dovitinib (**E**). Data are presented as mean ± SEM. ** *p* < 0.005, *** *p* < 0.001 vs. control; ^##^ *p* < 0.005, ^###^ *p* < 0.001 vs. FGF2.

We next studied the neurotrophin BDNF and its receptor TrkB, which are known to play an important role in myelination [29,30]. Application of dovitinib resulted in an increase in the number of BDNF^+^ cells (*p* = 0.005) (Figure 4A,B) and TrkB^+^ cells (*p* < 0.001) (Figure 5A,B). Treatment with AZD4547, however, increased only the number of TrkB^+^ (*p* = 0.002) (Figure 5A,B) but not BDNF^+^ cells (Figure 4A,B). Dovitinib caused an upregulation of BDNF and TrkB proteins (*p* < 0.001) (Figure 4D,E and Figure 5D,E). AZD4547 did not affect protein levels of TrkB or BDNF (Figure 4D,E and Figure 5D,E). In contrast to these findings, mRNA encoding for *BDNF* (*p* < 0.001) was increased by AZD4547 (Figure 4C). Furthermore, *NTRK2* mRNA expression was elevated after AZD4547 treatment (*p* < 0.001). Treatment with dovitinib did not alter mRNA levels of *BDNF* or *NTRK2*.

### 3.6. Expression of PLP and CNPase in OLN-93 Cells Is Increased by FGFR Inhibition

**Figure 6:**
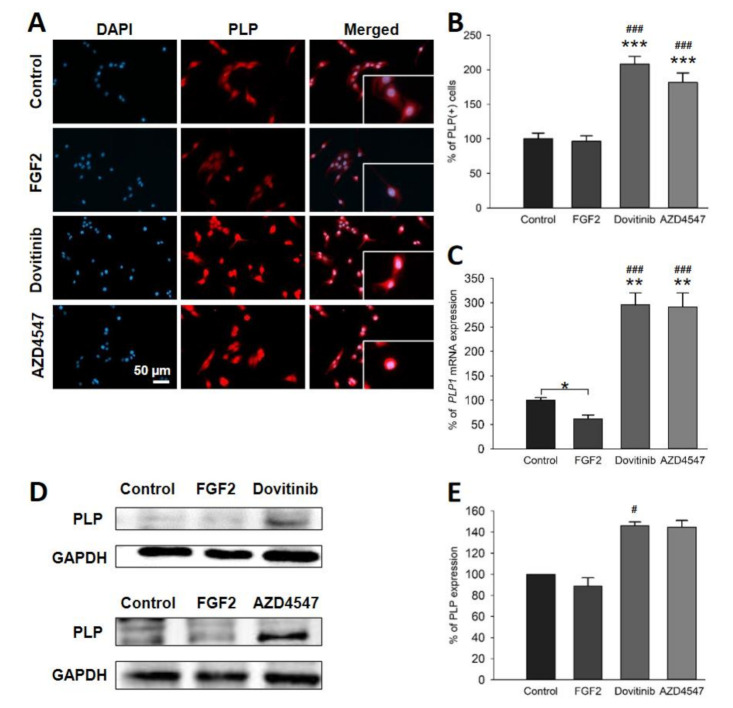
Distribution of PLP^+^ cells (**A**,**B**), PLP mRNA expression (**C**) and its amount of total protein (**D**,**E**). Representative images for immunofluorescence staining (**A**). Quantification of fluorescence revealed doubled PLP^+^ cell numbers after treatment with the FGFR inhibitors (**B**). mRNA expression of PLP (*PLP1*) was increased by treatment (**C**). Representative Western blot images of PLP analysis (**D**). PLP protein was increased by dovitinib (**E**). Data are presented as mean ± SEM. * *p* < 0.05, *** *p* < 0.001 vs. control; ^#^ *p* < 0.05, ^###^ *p* < 0.001 vs. FGF2.

**Figure 7:**
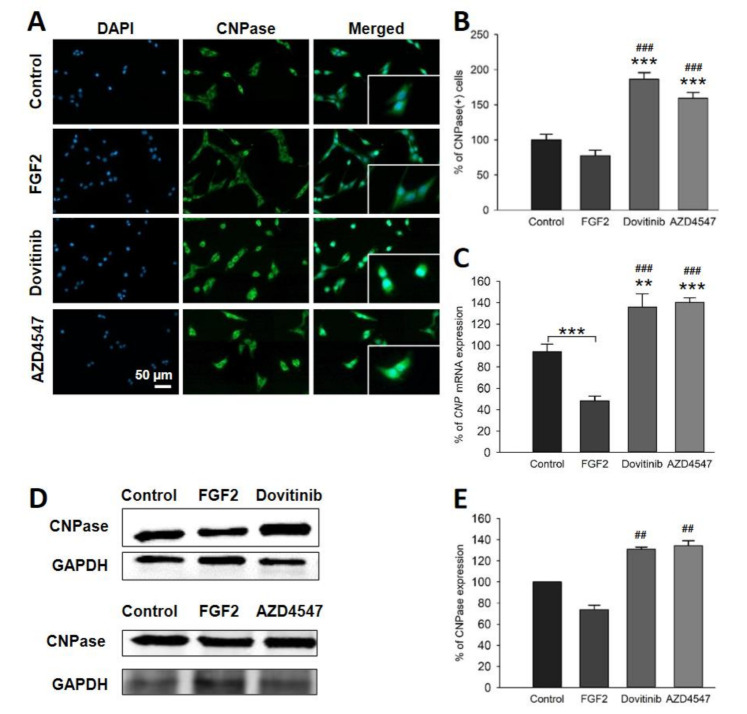
Immunofluorescence staining for CNPase^+^ cells (**A**,**B**), CNPase mRNA (**C**) and protein levels (**D**). Depicted representative sections showed increase in distribution and prevalence of CNPase (**A**). The number of CNPase^+^ cells was increased by more than 60% by AZD4547 or dovitinib treatment (**B**). mRNA analysis revealed decreased expression of CNPase (*CNP*) mRNA by FGF2; FGFR inhibitors enhanced mRNA levels (**C**). Representative Western blot bands for CNPase (**D**). Quantification of CNPase protein revealed increased expression by the inhibitors (**E**). Data are presented as mean ± SEM. ** *p* < 0.005, *** *p* < 0.001 vs. control; ^##^ *p* < 0.005, ^###^ *p* < 0.001 vs. FGF2.

To assess whether pharmacological inhibition of FGFR induces myelination, we investigated the expression of key myelin protein components [31]. Indeed, immunofluorescence staining showed a significant increase in PLP^+^ and 2′, 3′-Cyclic-nucleotide 3′-phosphodiesterase (CNPase)^+^ cells to both compounds (*p* < 0.001) (Figure 6A,B and Figure 7A,B) Further, Western blot analysis of dovitinib treated OLN-93 cells showed abundance for PLP compared to FGF2 (*p* = 0.026). In contrast, AZD4547 treated OLN-93 cells did not show changes in PLP protein levels (Figure 6D,E). *PLP1* mRNA was elevated by both inhibitors (AZD4547: *p* = 0.006; dovitinib: *p* = 0.005) compared to control and FGF2 (*p* < 0.001) (Figure 6C). In addition, CNPase protein levels were also increased by both inhibitors versus FGF2 (AZD4547: *p* = 0.03; dovitinib: *p* = 0.018) (Figure 7D,E). Similarly, comparative qRT-PCR showed increased mRNA levels of *CNP* for AZD4547 and dovitinib (AZD4547: *p* < 0.001; dovitinib: *p* = 0.002), and FGF2 (*p* < 0.001). Conversely, FGF2 treatment of oligodendrocytes significantly reduced mRNA levels of both *PLP* (*p* = 0.043) and *CNP* (*p* < 0.001) (Figure 6C and Figure 7C).

### 3.7. Expression of the Myelin Inhibitory Genes SEMA3A and TGFß Is Reduced after FGFR Inhibition by AZD4547

**Figure 8:**
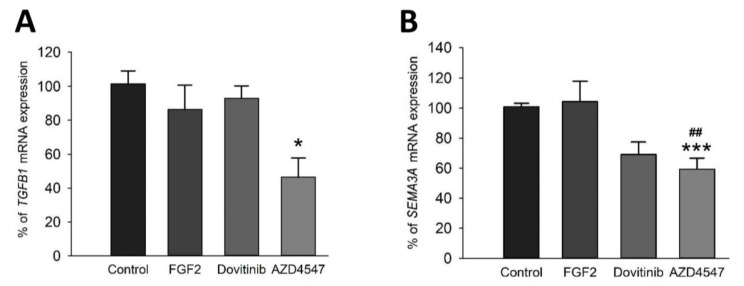
mRNA levels of TGFβ1 and SEMA3A. Expression of TGFβ (**A**) and SEMA3A (**B**) mRNA was reduced by AZD4547. While dovitinib and FGF2 did not regulate TGFβ (*TGFB1*) mRNA (**A**), AZD4547 reduced TGFβ mRNA expression (**A**) as well as mRNA levels of SEMA3A (*SEMA3A*) (**B**). Data are presented as mean ± SEM. * *p* < 0.05, *** *p* < 0.001 vs. control; ^##^ *p* < 0.005 vs. FGF2.

Semaphorin 3A (SEMA3A) is one of the factors known to suppress (re)myelination by impeding OPC migration [32]. Transforming growth factor β (TGFβ) has ambivalent roles in demyelination. We, therefore, assessed their expression after FGFR inhibition on mRNA levels using comparative qRT-PCR. The analysis revealed an abating effect of AZD4547 on mRNA levels of *TGFB1* (vs. control: *p* < 0.024) as well as *SEMA3A* (vs. FGF2: *p* = 0.007; vs. control: *p* < 0.001) (Figure 8A,B). Dovitinib did not regulate *TGFB1* gene expression, there was a strong trend towards downregulation of *SEMA3A* (Dovitinib vs. control: *p* = 0.062) (Figure 8A,B).

## 4. Discussion

There is increasing evidence for a role of FGF/FGFR signaling in the pathogenesis of MS and its disease model EAE. We recently showed that OL-specific deletion of either *FGFR1* or *FGFR2* resulted in a less severe disease course in MOG_35–55_-induced EAE [16,17]. Furthermore, detrimental effects of FGFR signaling in OLs on inflammation, myelin and axons were found in this disease model, highlighting the importance for the investigation of FGFR signaling in these myelin-producing cells. Therefore, to extend our understanding of FGFR signaling in oligodendrocytes, we studied the effects of pharmacological inhibition of FGFR in OLN-93 cells using the tyrosine kinase inhibitors dovitinib and AZD4547. We propose that the characterization of effects caused by FGFR inhibition in OLs can be a step towards the development of therapeutics abrogating impaired remyelination in MS.

To date, research on the function of FGFRs in oligodendrocytes has been done in knockout mice. The studies revealed that activation of ERK and Akt is central to the function of OPCs and OLs. These signaling proteins take part in pathways involved in proliferation and differentiation programs including process outgrowth and myelination [33,34,35,36,37]. Thus, ERK and Akt are considered to be important cellular actors in the OL cell cycle [38,39] and presumably the most important signaling molecules downstream of FGFRs [40]. Furthermore, FGFR inhibitors applied to FGFR^+^ cancer cells reduce the downstream signal transducers pERK and pAkt [19,41,42,43]. However, inhibition of the FGFR pathway specifically in OLs has not been explicitly studied yet. In the present in vitro experiments, both inhibitors of FGFRs reduced the proliferation of OLN-93 cells. Accordingly, FGFR inhibitors exert anti-proliferative effects in tumor cells [44,45] and vice versa, FGFs increase proliferation in various cells including OPCs [46,47,48,49]. This anti-proliferative effect of FGFR inhibition in OLN-93 cells was accompanied by a significant reduction of pErk and pAkt expression. There is considerable evidence that ERK and Akt are promoters of myelination [33,34,35,39]. Crosstalk between these two through mTOR signaling [34,36], as well as activation and inhibition of other pathways are probably involved in myelination and differentiation of OLs. In EAE, OL-specific deletion of *FGFR1* and *FGFR2* resulted in an upregulation of pAkt in the chronic phase of EAE. Since ERK/Akt levels were not changed in the acute phase of EAE, at a time point when beneficial effects of *FGFR* deletion were already present, activation of other pathways may account for the effects on myelin [16,17]. In this in vitro study, inhibition of FGFRs in OLs resulted in reduced phosphorylation of both direct downstream targets ERK and Akt. Despite the reduction of key signal proteins, an increase in myelin expression was observed, suggesting that in OLN-93 cells alternative supplementary or separate promyelinating pathways exist.

An alternative pathway modulating myelination may involve BDNF/TrkB signaling. In EAE, deletion of *FGFR1* in OL resulted in an upregulation of BDNF/TrkB and less myelin loss [16]. BDNF increases inflammation in the CNS, while acting as a protective factor for axons in EAE [50]. Its deletion accordingly leads to more severe clinical activity, reduced axonal density and increased demyelination in the acute phase of EAE [51]. Thus, a neuroprotective effect of BDNF/TrkB signaling in an immune-dependent demyelinated setting, such as in EAE, supports the concept of neuroprotective autoimmunity [52]. Furthermore, administration of a TrkB agonist resulted in neuroprotective effects in EAE, including decreased OL loss and inflammation, less demyelination and increased remyelination [53]. A promyelinating effect of BDNF/TrkB signaling also seemed to be present in an oligodendrocyte monoculture setting without immune cells. Findings from our in vitro experiments show that FGFR inhibition in OLN-93 cells inhibits canonical downstream signal transducers, while simultaneously activating the BDNF/TrkB pathway, that was previously considered to be independent. Taken together, reduced ERK/Akt expression associated with increased BDNF/TrkB levels suggest that OLs are capable of compensatory responses following FGFR inhibition.

In agreement with our hypothesis, inhibition of FGFR resulted in increased expression of myelin-specific proteins. Promotion of cell cycle switches in mature OLs with potential for myelination may take place. Even though FGFs are required for developmental myelination and may enhance proliferation of OPCs [49,54], their effects vary dependent on the microenvironment, presence of other cells, and the differentiation state of OLs. Concordantly with our results from cell-specific *FGFR* knockout studies in EAE [16,17], in vitro data support the view that inhibition of FGFR signaling in OLs results in enhanced myelination. Furthermore, the inhibitor of remyelination SEMA3A was reduced in OL-specific *FGFR1* and *FGFR2* knockout mice in EAE [16,17]. SEMA3A has not only been suggested to inhibit remyelination, it may also modulate immune responses by balancing T cell inflammation [32]. TGFβ, which has several cell-specific immunomodulatory roles, exerts anti-inflammatory effects on the microglia in the CNS [55,56]. Taken together, FGFR inhibition in OLN-93 cells does not only result in an increase of myelin proteins, it also decreases the expression of myelin inhibitors. Thus, inhibition of FGFR signaling in OLs induces myelination and intrinsically decreases inhibition of myelination.

The effects of the two inhibitors used in this study are similar but with different mechanisms. AZD4547 altered cell responses on the genetic level. It distinctly lowered the amount of FGFR1 mRNA, increased BDNF and TrkB mRNA and, more subtly, the *CNP* mRNA level in comparison to dovitinib. AZD4547 reduced myelin inhibitors TGFβ and SEMA3A. Surprisingly, alteration of expression patterns did not always result in higher protein expression. Here, dovitinib treatment resulted in distinct effects on downstream signaling (pERK, pAkt) compensatory pro-myelinating signals (BDNF, TrkB) and a slightly higher effect on myelin proteins. However, none of the differences between the two substances were statistically significant—except for a difference in mRNA levels of TrkB. The trend towards stronger action by dovitinib may point towards the relevance of other tyrosine kinases inhibited by dovitinib. These include VEGFRs, c-kit, colony stimulating factor 1 receptor (CSF1R), FMS-like tyrosine kinase 3 (FLT3) and PDGFRs [57]. CSFR1 is also inhibited by AZD4547 at a similar IC_50_ and will thus not explain the differences. All of the above-mentioned receptors are presumably expressed by oligodendrocytes at least either after injury or in progenitor states [58,59,60,61,62]. It is conceivable, that the effects of dovitinib are enhanced by its lower selectivity. Especially PDGFRs in mature OLs may be important for the enhanced response in the presence of dovitinib, as this receptor acts analogously to FGFRs [63,64].

The in vitro results from this study may be difficult to transfer to the CNS, where various cell types interact. However, EAE studies demonstrate that FGF/FGFR signaling affects myelination. Furthermore, most of the data from MS and demyelinating models show that FGFs accumulate in demyelinated lesions [13,14,65,66]. In EAE, effects of FGFR signaling are different between disease phases, but FGFR signaling is detrimental in the pathogenesis of EAE [16,17]. FGF2 and FGF9 were downregulated in the chronic phase of EAE in OL-specific *FGFR2* knockout mice [17]. MS research has focused on ligands rather than receptors. FGF2 levels in the CSF are elevated in patients [15] and FGF2^+^ microglia are present in active MS lesions [13]. FGF9 is increased primarily in OLs in active as well as in the boundary zone of chronic active lesions suggesting that it blocks myelination [14]. In contrast, it has been proposed that FGFs act as recruiting factors for OPCs thus promoting remyelination. Taken together, FGFs may initially be conducive to recruitment and proliferation, but their continued presence may inhibit remyelination.

Both AZD4547 and dovitinib led to a significant decrease of FGFR1^+^ cells upon inhibition of the receptor. Other than expected, OLN-93 cells showed no response to FGFR1 inhibition as explained by a proposed negative feedback loop that upregulates FGFR1 expression. Further, FGFR1 could be either post-transcriptionally degraded (dovitinib) or its mRNA expression decreased (AZD4547), suggesting a double negative feedback loop. In fact, this scenario has been described earlier, in which FGFR1 mRNA levels in OPCs decline after exposure to FGF2 [67]. However, our results showed a reverse effect to the absence of FGFR1-dependent signaling. This may underline the relevance of high abundance of FGF2 in a disease state such as EAE and MS, where FGF2 will continuously activate the signaling pathway which is otherwise down-regulated by auto-negative feedback. In this scenario, continuously increased levels of FGF2 from various sources activate the cellular pathway that is otherwise heavily confined by self-regulatory mechanisms, thus leading to detrimental effects for remyelination.

In summary, we showed that pharmacological inhibition of FGFRs successfully disrupted the FGFR signaling cascade with alterations in intrinsic cellular responses and myelin production. It is conceivable that decreased phosphorylation of ERK and Akt induces an upregulation of BDNF/TrkB signaling leading to enhanced production of myelin-specific proteins. Considering that inhibition of FGFRs is currently being used in various cancer trials, these tyrosine kinase inhibitors should be administrated in preclinical studies to evaluate their potential for remyelination and, if successful, applied in MS research trials.

## Data Availability

The data presented in this study are available on request from the corresponding author.

## Supplementary Materials

The following are available online at https://www.mdpi.com/article/10.3390/cells10061318/s1, Table S1. List of antibodies used in this study, Table S2. List of primers used in this study.

## Abbreviations

BDNF	Brain derived neurotrophic factor
CNPase	2′, 3′-cyclic-nucleotide 3′-phosphodiesterase
CNS	Central nervous system
CNTF	Ciliary neurotrophic factor
CSF	Cerebrospinal fluid
DMEM	Dulbecco’s Modified Eagle Medium
DMSO	Dimethyl sulfoxide
EAE	Experimental autoimmune encephalomyelitis
(p)ERK	(Phosphorylated) extracellular signal-regulated kinase
FGF	Fibroblast growth factor
FGFR	Fibroblast growth factor receptor
IGF	Insulin-like growth factor
LDH	Lactate dehydrogenase
MS	Multiple sclerosis
OL	Oligodendrocyte
OPC	Oligodendrocyte progenitor cell
PI3K	Phosphoinositide 3-kinase
PLP	Proteolipid protein
PDGF	Platelet derived growth factor
PDGFR	Platelet-derived growth factor receptor
SEM	Standard error of the mean
SEMA3A	Semaphorin 3A
TGFβ	Transforming growth factor β
TrkB	Tropomyosin receptor kinase B
VEGF	Vascular endothelial growth factor
