# Supplementary material for: Effects of FGFR Tyrosine Kinase Inhibition in OLN-93 Oligodendrocytes
Source: Cells. 2021 May 25;10(6):1318. doi: 10.3390/cells10061318 (PMC8228431; doi:10.3390/cells10061318)
Supplement: Supplementary file 1 [file cells-10-01318-s001.zip › cells-1193239-supplementary.pdf]

Table S1. List of antibodies used in this study

Antigen Name	Host	Mol. Weight (kDa)	Dilution	Method	Catalogue Nr	Manufacturer
Anti-Fgfr1	Rabbit	48-140	1:500	IF	SC-57132	Santa Cruz Biotechnology, Paso Robles, CA, USA
Anti-p-Akt	Rabbit	60	1:500	WB, IF	4060s	Cell Signalling Technology, Danvers, MA, USA
Anti-p-ERK	Rabbit	42, 44	1:500	WB, IF	4370s	Cell Signalling Technology, Danvers, MA, USA
Anti-TrkB	Mouse	95-145	1:100	WB, IF	SC-377218	Santa Cruz Biotechnology, Paso Robles, CA, USA
Anti-pro BDNF	Mouse	14	1:500	WB, IF	SC-65514	Santa Cruz Biotechnology, Paso Robles, CA, USA
Anti-PLP	Mouse	30	1:1000	WB, IF	SC-23570	Santa Cruz Biotechnology, Paso Robles, CA, USA
Anti-CNPase	Mouse	46	1:500	WB, IF	SC-166019	Santa Cruz Biotechnology, Paso Robles, CA, USA
Anti-GAPDH	Mouse	37	1:2500	WB	SC-365062	Santa Cruz Biotechnology, Paso Robles, CA, USA
Goat anti-rabbit	Goat	Secondary Ab	1:1000	WB	SC-2004	Santa Cruz Biotechnology, Paso Robles, CA, USA
Donkey anti-mouse	Donkey	Secondary Ab	1:1000	WB	SC2096	Santa Cruz Biotechnology, Paso Robles, CA, USA
Donkey anti-goat	Donkey	Secondary Ab	1:2500	WB	SC2020	Santa Cruz Biotechnology, Paso Robles, CA, USA
Goat anti-rabbit Alexa Fluor 488	Goat	Secondary fluorescent Ab	1:500	IF	A-11070	Invitrogen, Carlsbad, CA, USA
Goat anti-mouse Alexa Fluor 488	Goat	Secondary fluorescent Ab	1:500	IF	A-21121	Invitrogen, Carlsbad, CA, USA
Rabbit anti-goat Alexa Fluor 594	Rabbit	Secondary fluorescent Ab	1:500	IF	A-11080	Invitrogen, Carlsbad, CA, USA

Table S2. List of primers used in this study

Gene	Primer name		Primer Sequence (5´ → 3´)	Annealing temperature	Manufacturer
GAPDH	GAPDH	forward	AGT GCC AGC CTC GTC TCA TA	59.4 °C	Eurofins Genomics, Ebersberg, BY, Germany
		reverse	GGT AAC CAG GCG TCC GAT AC	61.4 °C	
FGFR1	FGFR1	forward	CGT GCC TGT GGA AGA ACT TT	57.3 °C	
		reverse	CCG CAT CAT CAT GTA CAG CTC	59.8 °C	
BDNF	BDNF	forward	GGA CCA GGA GCG TGA CAA	58.2 °C	
		reverse	TCT CAC CTG GTG GAA CTC AG	59.4 °C	
NTRK2	TrkB	forward	CAC ACA CAG GGC TCC TTA	56.0 °C	
		reverse	AGT GGT GGT CTG AGG TTG G	58.8 °C	
PLP1	PLP	forward	GTG TTC TCC CAT GGA ATG CT	57.3 °C	
		reverse	TGA AGG TGA GCA GGG AAA CT	57.3 °C	
CNP	CNPase	forward	CCA ACA GGA TGT GGT GAG GA	59.4 °C	
		reverse	AGC TGT CTT GGG TGT CAC AA	57.3 °C	
SEAM3A	SEMA3A	forward	TCT GTA GGT AGC CAC ATT CGAT	58.4 °C	
		reverse	TCT GGA AAC TCC TGA GGC AAG	59.8 °C	
TGFβ1	TGFβ	forward	CTG CTG ACC CCC ACT GAT AC	61.4 °C	
		reverse	AGC CCT GTA TTC CGT CTC CT	59.4 °C	
